# Protocol for fabricating a vascularized bile duct-on-a-chip

**DOI:** 10.52601/bpr.2025.240069

**Published:** 2025-12-31

**Authors:** Chaoyang Song, Mengqi Zhu, Rebecca G. Wells, Yu Du

**Affiliations:** 1 Center for Biomechanics and Bioengineering, Beijing Key Laboratory of Engineered Construction and Mechanobiology and Key Laboratory of Microgravity (National Microgravity Laboratory), Institute of Mechanics, Chinese Academy of Sciences, Beijing 100190, China; 2 School of Engineering Sciences, University of Chinese Academy of Sciences, Beijing 100049, China; 3 Medical Science and Technology Innovation Center, Shandong First Medical University & Shandong Academy of Medical Sciences, Jinan 250117, China; 4 Department of Medicine, Perelman School of Medicine, University of Pennsylvania, Philadelphia, PA 19104, USA; 5 NSF Science and Technology Center for Engineering MechanoBiology, University of Pennsylvania, Philadelphia, PA 19104, USA; 6 Department of Bioengineering, School of Engineering and Applied Sciences, University of Pennsylvania, Philadelphia, PA 19104, USA

**Keywords:** Organ-on-a-chip, Cholangiopathy, Vascularized, Cholestatic liver diseases

## Abstract

The care of patients with cholestatic liver diseases such as primary sclerosing cholangitis (PSC) is challenging, partly due to the lack of knowledge of disease pathogenesis and suitable *in vitro* models for disease modeling and drug screening. Although the pathogenesis of cholestatic liver diseases like PSC remains unknown, the importance of the vascular-biliary interface is clear. Cholangiocyte injury not only impairs barrier function such that bile leaks and damages periductal tissue, but also activates cholangiocytes to secret pro-inflammatory and pro-fibrogenic mediators to stimulate immune cells and mesenchymal cells, ultimately causing damage to the liver. Here we describe a detailed protocol for fabricating a human vascularized bile duct-on-a-chip (VBDOC) that consists of a vascular channel, biliary channel, and neighboring mesenchymal cells in a collagen gel that models the vascular-biliary interface structurally and functionally in three dimensions. This device is notable in maintaining cholangiocyte polarity and barrier function, recapitulating physiological functions and responses of the large bile ducts, and enabling manipulation of components of the mechanical microenvironment such as matrix stiffness and shear flow in the lumens. This practical workflow could help researchers manufacture the VBDOC in their own labs and apply it to studies of various cholestatic liver diseases.

## INTRODUCTION

Cholestatic liver diseases carry significant morbidity and mortality and remain a major challenge in hepatology (Karlsen *et al.* 2017). Treatment options are limited and liver transplantation is often the only option, especially for diseases such as primary sclerosing cholangitis (PSC) (Lazaridis *et al.* 2015). The development of new therapeutic approaches is hampered by the lack of knowledge of disease pathogenesis and suitable *in vitro* models for disease modeling and drug screening (Brevini *et al.* 2020; Ji *et al.* 2017).

Although the pathogenesis of cholestatic liver diseases like PSC remains unknown, the importance of the vascular-biliary interface has been shown by previous studies (Banales *et al.* 2019; Pinzani *et al.* 2018; de Krijger *et al.* 2019). Cholangiocyte injury could, on the one hand, impair barrier function such that bile leaks and damaged periductal tissue (Merlen *et al. *2020), or, on the other hand, activate cholangiocytes to secret pro-inflammatory and pro-fibrogenic mediators to stimulate immune cells and mesenchymal cells ultimately causing damage to the liver (Pinzani *et al.* 2018; de Krijger *et al.* 2019; Kunzmann *et al.* 2020; Dyson *et al.* 2018). The microenvironment of the vascular-biliary interface is complex as potential mechanisms involve multiple factors, including different types of cells (cholangiocytes, immune cells, endothelial cells and mesenchymal cells), extracellular matrix (ECM) remodeling, and mechanical forces (bile flow, blood flow, ductular pressure accumulation, tensile force of mesenchymal cells).

To model the complex microenvironment, features such as multicellular interactions, a tunable ECM and the integration of mechanical cues need to be incorporated (Brevini *et al.* 2020). Animal models are limited by interspecies variation and a limited ability to manipulate key parameters such as duct size, cell types, shear stress and ECM stiffness (Reich *et al.* 2021; Pollheimer *et al.* 2018). *In vitro* models such as organoids from primary human cells have emerged as a useful tool with high fidelity (Huch *et al.* 2015; Soroka *et al.* 2018; Lorent *et al.* 2015) but are still limited in capturing the tubular three-dimensional (3D) structure, fluid flow and interactions between different types of cells that characterize the *in vivo* setting. Organ-on-chip technology could model the physiological structure and functions of tissues and organs by using microfluidic devices for co-culturing various types of cells in 3D structures and recapitulating the physical environment, especially the presence of mechanical stimuli (Polacheck *et al. *2019).

Based on two microfluidic devices we previously developed, a bile duct-on-a-chip (Du *et al.* 2020) and a liver sinusoidal chip (Du *et al.* 2017), we established a new *in vitro* platform, a vascularized bile duct-on-a-chip, or VBDOC, that is based on organoid technology (Tysoe *et al.* 2019) and includes multiple cell types (Du *et al.* 2023). This device is notable in that cholangiocyte polarity and barrier function are maintained, and initial studies suggest that it is a good model of many of the physiological functions and responses of the large bile ducts. Here we provide a detailed protocol for the fabrication and basic characterization of the VBDOC. We hope that this practical workflow will help researchers manufacture the VBDOC in their own labs and use it for studies of various cholestatic liver diseases.

## OVERVIEW OF THE PROTOCOL

**Figure 1:**
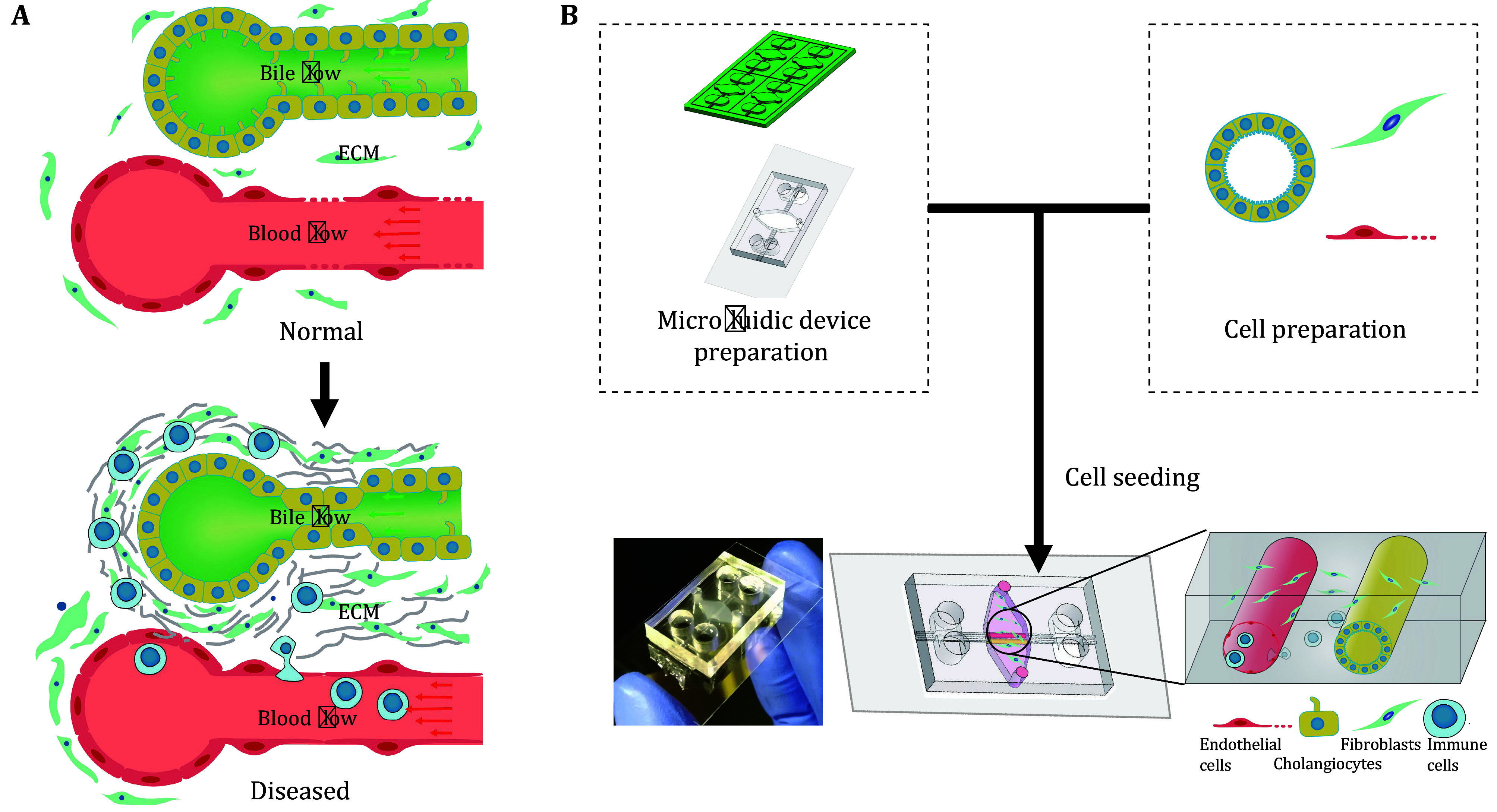
Overview of VBDOC fabrication. **A** Schematics of normal and diseased vascular-biliary interfaces highlighting increased matrix deposition and numbers of fibrogenic and inflammatory cells in the setting of disease. **B** Overview of the protocol. Part of this figure is modified from Du *et al*. (2023)

This protocol consists of three parts. The first introduces the design and fabrication of the microfluidic device. The second describes the generation and culture of cholangiocyte organoids, and the third provides a method to seed cells to form the VBDOC (Fig. 1).

### Design and fabrication of the microfluidic devices

The design of the chip is carried out using Auto CAD, and the microfluidic mold is manufactured with a high-resolution 3D printer. Microfluidic devices are fabricated by plasma bonding the coverslips and PDMS stamps produced through replica molding.

### Generation of organoids

Biliary organoids, used as cell sources in the device, can be generated from bile duct tissue and bile. Cholangiocytes can be obtained from fresh human tissue through mechanical scraping of the bile ducts or from bile collected by endoscopic retrograde cholangiopancreatography (ERCP). Organoids are generated by culturing the isolated cholangiocytes in Matrigel and can be passaged over 20 times (Tysoe *et al.* 2019).

### Cell seeding

Within the microfluidic devices, lumens within the fibroblast-embedded natural matrix are generated by gelling the matrix around needles. Cholangiocytes and human umbilical vein endothelial cells are seeded into the different channels and cultured into confluent and compact monolayers.

## MATERIALS, INSTRUMENTATION AND SOFTWARE

### Primary cells and cell lines

• Human cholangiocytes from tissue or bile

• Human umbilical vein endothelial cells (HUVECs, CC-2519, Lonza; or primary cells)

• Human gallbladder fibroblasts (HGBFs, 5430, ScienCell; or primary cells)

### Reagents

• Matrigel (356234, BD Biosciences)

• William’s E medium, no phenol red (A1217601, Invitrogen)

• Nicotinamide (N0636, Sigma-Aldrich)

• Sodium bicarbonate (S6014, Sigma-Aldrich)

• 2-phospho-L-ascorbic acid trisodium salt (49752, Sigma-Aldrich)

• Glucose (D9434, Sigma-Aldrich)

• Sodium pyruvate (P2256, Sigma-Aldrich)

• 4-(2-hydroxyethyl)-1-piperazineethanesulfonic acid (HEPES) (83264, Sigma-Aldrich)

• Insulin-transferrin-selenium + premix (ITS + premix) (354352, BD Biosciences)

• Dexamethasone (D4902, Sigma-Aldrich)

• Glutamax (35050061, Invitrogen)

• Penicillin and streptomycin (15140122, Thermo Fisher Scientific)

• EGF (236-EG, R&D Systems)

• R-spondin (4645-RS, R&D Systems)

• DKK-1 (5439-DK, R&D Systems)

• Y27632 (Y0503, Sigma-Aldrich)

• Cell recovery solution (354253, Corning)

• PDMS (Sylgard 184, Dow-Corning)

• Collagen type I, rat tail (356236, Corning)

• 10× DMEM medium (M0650, Sigma)

• NaOH

• NaHCO_3_

• Poly-L-lysine.

• Glutaraldehyde (A17876, Thermo Fisher Scientific)

• GFP lentivirus (17448, Addgene)

• DPBS

• 4% PFA (AAJ19943K2, Thermo Fisher Scientific)

• Triton X-100 (85111, Thermo Fisher Scientific)

• BSA (V900933, Sigma-Aldrich)

• Cellbanker 2 (ZENOAQ)

• TrypLE (Thermo Fisher Scientific)

• RPMI 1640 + Glutamax medium (61870036, Thermo Fisher Scientific)

• Fetal calf serum (FCS, 26010074, Thermo Fisher Scientific)

• Basal medium (BM): 10 mmol/L Nicotinamide, 17 mmol/L Sodium bicarbonate, 0.2 mmol/L 2-phospho-l-ascorbic acid trisodium salt, 14 mmol/L Glucose, 6.3 mmol/L Sodium pyruvate, 20 mmol/L HEPES, 2 mmol/L Glutamax, 1% ITS + premix, 1% Penicillin-Streptomycin, 0.1 µmol/L Dexamethasone in William’s E medium

• Complete cholangiocyte culture medium (CCCM): 10 mmol/L Nicotinamide, 17 mmol/L sodium bicarbonate, 0.2 mmol/L 2-phospho-l-ascorbic acid trisodium salt, 14 mmol/L glucose, 6.3 mmol/L Sodium pyruvate, 20 mmol/L HEPES, 2 mmol/L Glutamax, 1% ITS + premix, 1% Penicillin-Streptomycin, 0.1 µmol/L Dexamethasone, 20 ng/mL EGF, 500 ng/mL R-spondin, 100 ng/mL DKK-1 in William’s E medium

• Fibroblast Growth Medium kit (FGM, CC-3132, Lonza)

• Endothelial Cell Growth Medium kit (EGM, CC-3202, Lonza)

• Complete RPMI: 10% FCS, 1% P/S in RPMI 1640 + Glutamax medium

### Equipment

• Programmable hot plate (Thermo Fisher Scientific)

• Plasma etcher (Plasma Etch, United States)

• Vacuum pumps (Plantinum, United States)

• Rocker (Thermo Fisher Scientific)

• Leica confocal microscope (Leica Microsystems)

• Steel acupuncture needles (200 µm diameter; Seirin, Kyoto, Japan)

• Stereolithography 3D printer (Protolabs, United States)

• Ultrasonic cleaner

• Fume hood

• Desiccator

• Oven

• Nitrogen tank with air gun

• Centrifuge

• CO_2_ Incubator

### Software

• AutoCAD (Autodesk)

• Leica application suite X (Leica Microsystems)

## PROCEDURE

### PDMS device manufacturing

#### Design and fabrication of the chip

##### Design and manufacture the mold

**Figure 2:**
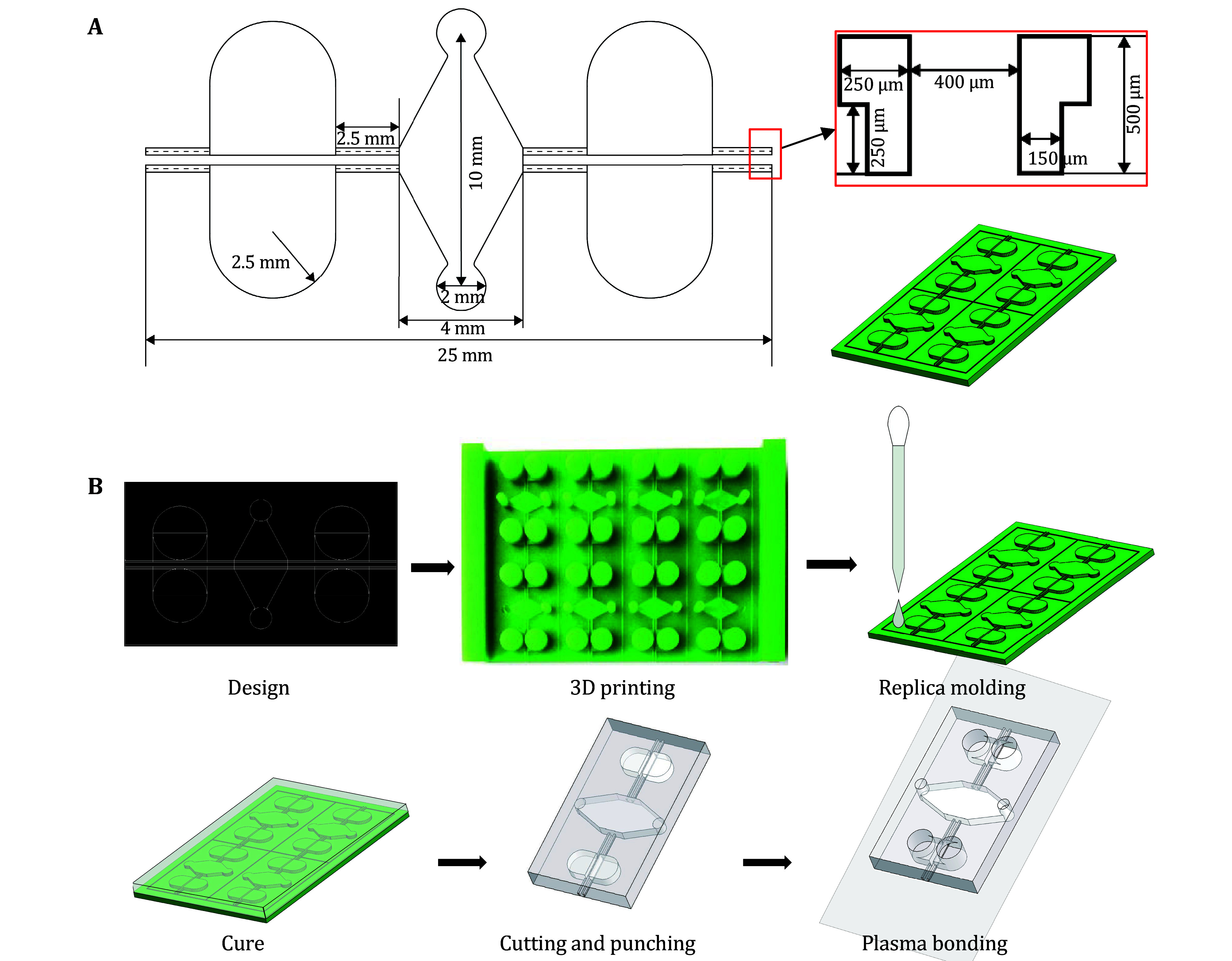
Microfluidic device manufacturing. **A** Detailed parameters of the design. The right top panel is the cross-sectional view of the scaffold structure and the right bottom panel is an overview of the 3D design. **B** Flow chart for microfluidic device fabrication. Scale bar: 200 µm

1 The mold is designed with AutoCAD; detailed parameters are shown in Fig. 2A.

2 The mold is manufactured with Microfine materials by a high-resolution 3D printer commercially.

##### Silanization

3 The 3D-printed mold is placed into a desiccator.

4 Add 20 µL tri-chloro(1H,1H,2H,2H-tridecafluoro-n-octyl)silane on a glass dish next to the mold in the desiccator, connect the desiccator to a vacuum pump and leave overnight.

#### PDMS device preparation

##### Preparation of PDMS mixture

1 Mix PDMS base and curing agent thoroughly in a 10:1 weight ratio. Then, place the mixture into a vacuum chamber and degas it until no air bubbles remain.

##### Replication of 3D printed mold

2 Pour the PDMS mixture into the mold.

3 Degas in the desiccator until there are no visible bubbles in the mixture.

4 Cure the PDMS fully at 75°C for 1.5 h.

5 Peel the PDMS gel carefully off the mold and use a razor blade to cut the cured PDMS gels into bricks of equal size (2 cm × 1.5 cm).

6 Form the reservoir ports using a 4-mm biopsy punch and the side ports using a 2-mm punch.

##### PDMS device assembly

7 Clean the PDMS device using adhesive tape and the coverslips by spraying with a nitrogen gun.

8 Apply the plasma treatment for 45 s to activate the PDMS surface groups. Immediately place the PDMS brick onto the coverslip and allow it to bond.

##### Surface treatment of the device

9 Inject 0.01% (*v*/*v*) poly-L-lysine (approximately 80 µL per device) into each chamber through the side port; incubate for 1 h at room temperature (RT).

10 Aspirate the poly-L-lysine and incubate with 0.5% (*v*/*v*) glutaraldehyde (approximately 80 µL per device) for 20 min.

11 Aspirate the glutaraldehyde and wash the device with dH_2_O three times.

12 Immerse the device in dH_2_O, sonicate for 30 min, and immerse in fresh dH_2_O overnight.

13 Rinse the devices with 70% ethanol for 30 min.

14 Incubate 200 µm steel acupuncture needles in dH_2_O-0.1% (*w*/*v*) BSA for 1 h.

15 Air-dry the device using a nitrogen gun.

16 Insert the needles from opposite directions through the channels (formed by the mold) until the tips of the needles reach the second reservoir port.

17 Sterilize the device under UV for 20 min.

### Cell preparation (Fig. 3)

**Figure 3:**
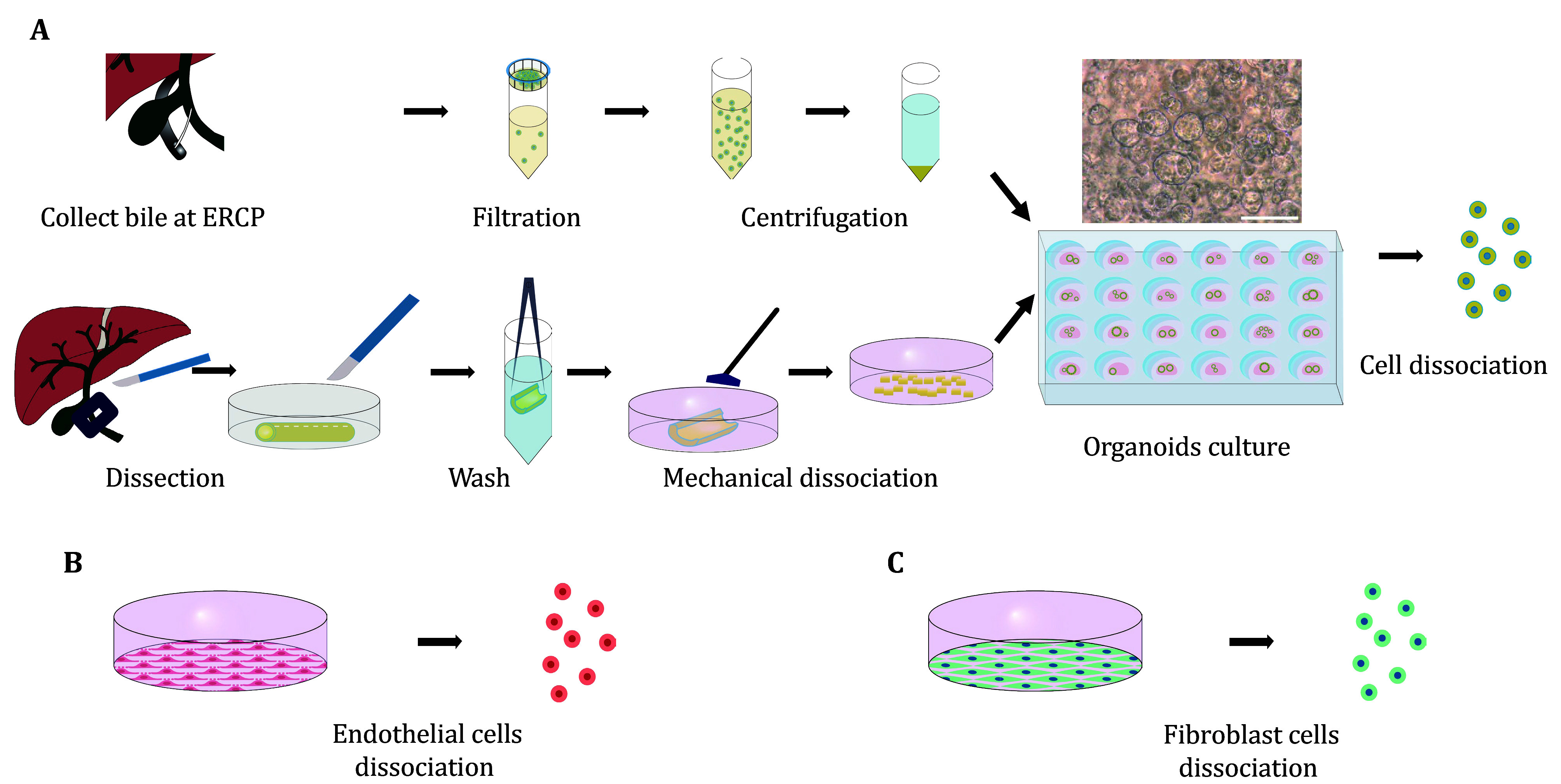
Preparation of cells for seeding. **A** Cholangiocyte isolation, organoid culture and preparation from bile duct tissue and bile. **B** Preparation of endothelial cells from tissue culture dish. **C** Preparation of fibroblast cells from the tissue culture dish. Part of this figure is modified from Du *et al*. (2023)

#### Isolation of cholangiocytes

##### Cholangiocytes from extrahepatic bile duct tissue

1 Obtain extrahepatic bile ducts in accordance with conditions defined by the local institutional review board (IRB).

2 Place the collected bile duct tissue into ice-cold BM and store at 4°C until further processing is possible.

3 Using a scalpel and forceps, longitudinally bisect the bile duct to expose the entire lumen.

4 Transfer the bile duct tissue into a 50 mL centrifuge tube containing DPBS to remove excess bile. Repeat the washing twice, using a fresh centrifuge tube each time.

5 Transfer the bile duct tissue into a fresh dish and add the BM until the tissue is fully submerged.

6 Gently scrape the epithelial layer from the luminal side of the duct using a scalpel until the luminal side appears smooth.

7 Transfer the scraped cells and medium to a new centrifuge tube and centrifuge at 444*g* for 4 min at RT to collect the cells.

8 Discard the supernatant.

9 Resuspend the cell pellet in 10 mL BM to wash the cells.

10 Centrifuge at 444*g* for 4 min at RT.

11 Discard the supernatant, obtaining a cell pellet.

##### Cholangiocytes from bile

1 During an ERCP procedure, following the guidelines of the local IRB, collect 2–5 mL bile from the midportion of the common bile duct (Soroka *et al.* 2018).

2 Dilute 1:10 in DPBS.

3 Use a 10 mL sterile pipette to mix the bile well and disperse particulate debris.

4 Filter the diluted bile through a 70 µm filter to remove insoluble material.

5 Centrifuge at 444*g* for 4 min. Discard the supernatant.

6 Resuspend the pellet in DPBS. Centrifuge at 444*g* for 4 min. Discard the supernatant.

7 Resuspend the pellet in BM. Centrifuge at 444*g* for 4 min.

8 Discard the supernatant and save the cell pellet.

##### Generation and culture of organoids

1 Cholangiocyte seeding.

(A) Prewarm a 24-well plate with a hot plate at 37°C for 30 min.

(B) Resuspend the bile and tissue cholangiocyte pellets in a mixture of 66% Matrigel and 33% CCCM.

(C) Plate 300 μL of the cell suspension per well of a 24-well plate. Incubate the plate at 37°C for 30 min to allow the Matrigel to solidify.

(D) After the Matrigel has been solidified, add 1 mL CCCM with 10 μmol/L Y27632 per well.

(E) Change the medium to CCCM alone after two days.

(F) Change the medium every two days thereafter.

(G) Organoids will form within three to ten days.

2 Organoid passage.

(H) Passage the organoids every five days, as follows.

( I ) Remove the medium.

( J ) Depolymerize the Matrigel with 1 mL cold cell recovery solution per well. Wash wells with new 500 μL cold cell recovery solution [Note: Eject cell recovery solution forcefully in order to fully disrupt the Matrigel].

(K) Transfer the organoids into a 15 mL centrifuge tube, and maintain the tube on ice for 30 min.

(L) Add cold HBSS up to 14 mL and centrifuge at 444*g* for 4 min at 4°C.

(M) Discard supernatant.

(N) Use 200 µL cold HBSS to mechanically disperse the organoids by pipetting up and down 40 to 50 times. Then add HBSS to 1 mL.

(O) Centrifuge at 444*g* for 4 min at 4°C. Discard supernatant.

(P) Resuspend the pellet in a mixture of 66% Matrigel and 33% CCCM for the next seeding.

3 Organoid cryopreservation.

(Q) After Step 16, organoids can be cryopreserved if desired using Cellbanker 2.

#### Human gallbladder fibroblast culture (HGBF) preparation

##### Culture

1 Plate 5 × 10^5^ cells of HGBFs in a T-25 flask in 5 mL FGM. Incubate at 37°C with 5% CO_2_.

2 Change the medium every two to three days.

##### Passage

3 Passage the cells when the culture reaches 95% confluency.

4 Place the FGM, TrypLE, and DPBS in a 37°C water bath.

5 Add 2 mL DPBS to rinse the cells.

6 Add 2 mL TrypLE, and incubate at 37°C for 1 min to dissociate the cells.

7 Gently tap the side of the flask to dislodge cells from the surface.

8 Check under a microscope to ensure that all cells have detached.

9 Add 2 mL FGM to neutralize the TrypLE.

10 Centrifuge at 300*g* for 5 min. Discard the supernatant.

11 Resuspend the pellet in FGM for seeding in new T-25 flasks.

#### HUVEC preparation

##### Culture

1 Plate 1 × 10^5^ HUVEC cells in a T-25 flask in 5 mL of EGM. Incubate at 37°C with 5% CO_2_.

2 Change the medium every two days.

##### Passage

3 Passage the cells when the culture reaches 75% confluency.

4 Place EGM, TrypLE, and DPBS in a 37°C water bath.

5 Add 2 mL DPBS to rinse the cells.

6 Add 2 mL TrypLE, and incubate at 37°C for 3 min to dissociate the cells.

7 Gently tap the side of the flask to dislodge cells from the surface.

8 Check under a microscope to ensure that all cells have been detached.

9 Add 2 mL EGM to neutralize the TrypLE.

10 Centrifuge at 200*g* for 5 min. Discard the supernatant.

11 Resuspend the pellet in EGM for seeding in new T-25 flasks.

### Cell seeding (Fig. 4)

**Figure 4:**
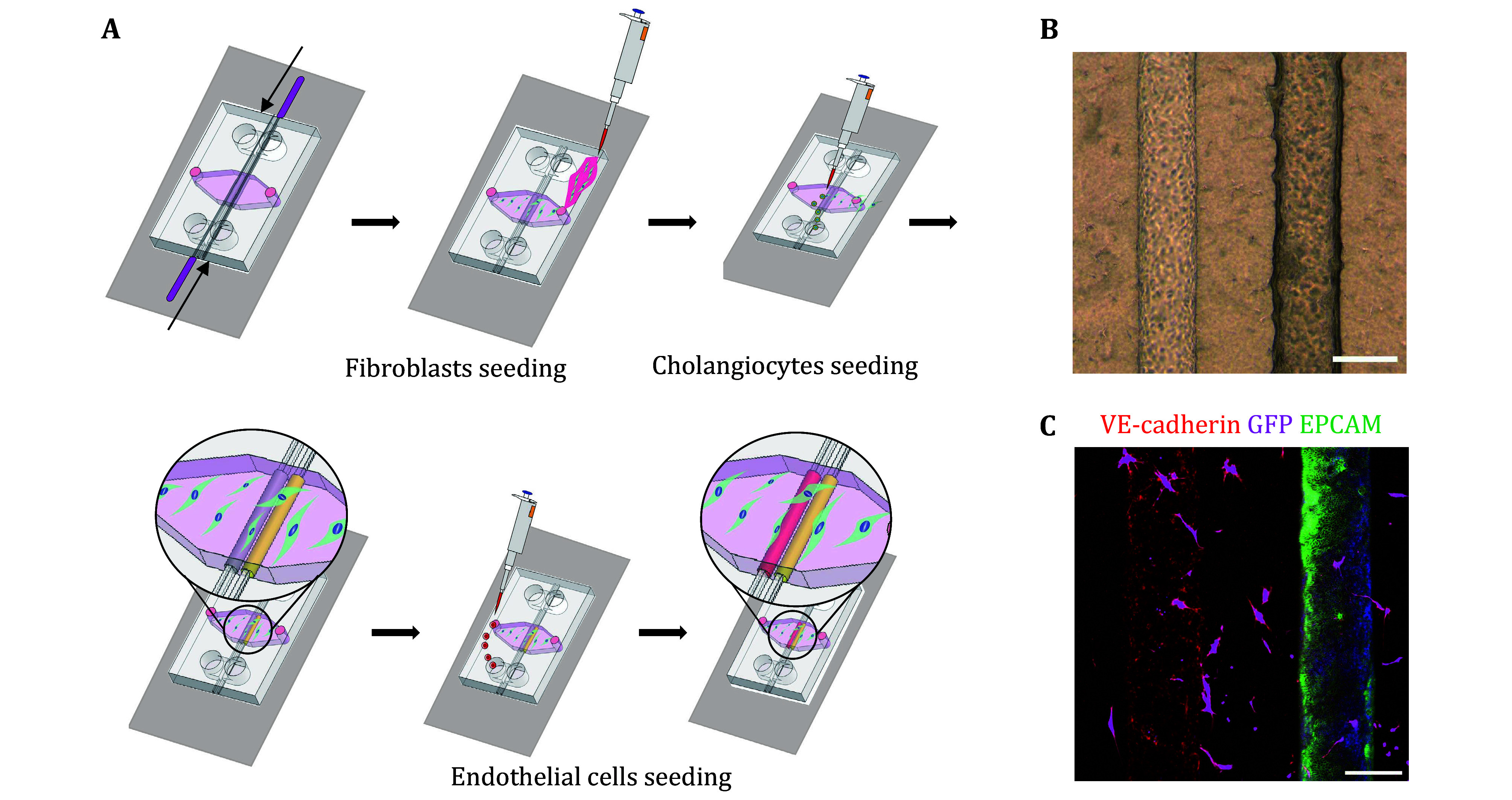
Cell seeding of the VBDOC. **A** Flow chart of the seeding process. **B** Bright field images of the cells in the VBDOC. Scale bars: 200 μm. **C** Representative immunofluorescence images of endothelial cells (VE-cadherin, red), fibroblasts (transfected GFP, magenta) and cholangiocytes (K19, green) in the VBDOC. Scale bars: 200 μm. This figure is modified from Du *et al*. (2023)

#### Preparation of collagen gel mixed with fibroblasts

1 1 Prepare a collagen solution at a concentration of 2.5 mg/mL using rat tail type I collagen, and keep it on ice. Add 10× DMEM medium, 10 mmol/L HEPES, 1 mol/L NaOH, and NaHCO_3_ (0.035% *w*/*v*) to adjust the pH to 7.0.

2 Trypsinize HGBF from culture dishes.

3 Resuspend the HGBF with the neutralized collagen solution at a density of 1.5 × 10^5^ cells/mL.

4 Inject the fibroblast/collagen mixture through the side ports of the device to fill the collagen gel chamber (about 70 µL per device). Note that the device will still contain the needles used to form channels.

5 Incubate the device, inverted, at 37°C for 20 min to allow the collagen to solidify.

6 Fill ports with fibroblast growth medium (about 120 µL per reservoir port) and incubate overnight.

7 Remove the needles, yielding two parallel channels through collagen. Seal the channel ends using vacuum grease.

#### Preparation of cholangiocyte channel

1 Prepare a cholangiocyte suspension at a density of 5 × 10^5^ cells/mL.

2 Inject 40 µL of the suspension into one reservoir port of the cholangiocyte channel and 30 µL into the other port of the same channel.

3 Incubate the device inverted at 37°C for 5 min to allow cholangiocytes to attach to the top surface of the channel.

4 Flip the device and allow cholangiocytes to attach to the bottom surface of the channel for 5 min at 37°C.

5 Remove cells in the reservoir ports by scraping with 100 μL tips.

6 Rinse the cholangiocyte channel with BM to remove nonadherent cells.

7 Fill the device with fresh CCCM supplemented with 10 µmol/L Y27632 (about 120 µL per port).

8 Incubate the device at 37°C with 5% CO_2_ on a rocker at 5 r/min for two days.

9 Switch the medium to CCCM without Y27632 and change the medium every two days until confluent monolayers have formed.

#### Preparation of blood vessel channel

1 Once the cholangiocyte monolayer reaches confluence (in about one week), prepare a HUVEC suspension at a density of 5 × 10^5^ cells/mL.

2 Inject 40 µL of the suspension into one reservoir port of the endothelial channel and 30 µL into the other port of the same channel.

3 Incubate the devices inverted at 37°C for 2 min to allow HUVECs to attach to the top surface of the channel.

4 Flip the device to allow HUVECs to attach to the bottom surface of the channel for 2 min at 37°C.

5 Scrape the cells in the reservoir ports with 100 μL tips.

6 Rinse with EGM to remove nonadherent cells.

7 Fill the reservoir ports of the endothelial channel with EGM and the cholangiocytes channel with CCCM (about 120 µL per port).

8 Maintain the device at 37°C with 5% CO_2_ on a rocker at 5 r/min until endothelial monolayers become confluent and cholangiocytes monolayers are confluent and compacted. As a control, static groups are maintained on a flat incubator shelf.

## ANTICIPATED RESULTS

After the completion of PDMS device manufacturing, microfluidic devices have been manufactured. After cell preparation, cholangiocyte organoids from tissue or bile, fibroblasts and endothelial cells have been obtained. After cell seeding, the VBDOC has been constructed, as shown in Fig. 1. Neighboring endothelial and biliary channels surrounded by fibroblast-embedded matrix will be within the chamber of the chip, as shown by the brightfield image in Fig. 4B and immunofluorescent image in Fig. 4C.

## Conflict of interest

Chaoyang Song, Mengqi Zhu, Rebecca G. Wells and Yu Du declare that they have no conflict of interest.

## References

[bBanales2019] (2019). Cholangiocyte pathobiology. Nat Rev. Gastroenterol hepatol.

[bBrevini2020] (2020). Tissue engineering of the biliary tract and modelling of cholestatic disorders. J Hepatol.

[bde2019] (2019). Return to sender: lymphocyte trafficking mechanisms as contributors to primary sclerosing cholangitis. J Hepatol.

[bDu2017] (2017). Mimicking liver sinusoidal structures and functions using a 3D-configured microfluidic chip. Lab Chip.

[bDu2023] (2023). Human vascularized bile duct-on-a chip: a multi-cellular micro-physiological system for studying cholestatic liver disease. Biofabrication.

[bDu2020] (2020). A bile duct-on-a-chip with organ-level functions. Hepatology (Baltimore, Md. ).

[bDyson2018] (2018). Primary sclerosing cholangitis. Lancet.

[bHuch2015] (2015). Long-term culture of genome-stable bipotent stem cells from adult human liver. Cell.

[bJi2017] (2017). Genome-wide association study of primary sclerosing cholangitis identifies new risk loci and quantifies the genetic relationship with inflammatory bowel disease. Nat Genet.

[bKarlsen2017] (2017). Primary sclerosing cholangitis – a comprehensive review. J Hepatol.

[bKunzmann2020] (2020). Monocytes as potential mediators of pathogen-induced T-helper 17 differentiation in patients with primary sclerosing cholangitis (PSC). Hepatology.

[bLazaridis2015] (2015). The cholangiopathies. Mayo Clin proc.

[bLorent2015] (2015). Identification of a plant isoflavonoid that causes biliary atresia. Sci Transl Med.

[bMerlen2020] (2020). TGR5-dependent hepatoprotection through the regulation of biliary epithelium barrier function. Gut.

[bPinzani2018] (2018). Pathogenesis of biliary fibrosis. Biochim Biophys Acta Mol Basis Dis.

[bPolacheck2019] (2019). Microfabricated blood vessels for modeling the vascular transport barrier. Nat Protoc.

[bPollheimer2018] (2018). Lysyl oxidase-like protein 2 (LOXL2) modulates barrier function in cholangiocytes in cholestasis. J Hepatol.

[bReich2021] (2021). Downregulation of TGR5 (GPBAR1) in biliary epithelial cells contributes to the pathogenesis of sclerosing cholangitis. J Hepatol.

[bSoroka2018] (2018). Bile-derived organoids from patients with primary sclerosing cholangitis recapitulate their inflammatory immune profile. Hepatol.

[bTysoe2019] (2019). Isolation and propagation of primary human cholangiocyte organoids for the generation of bioengineered biliary tissue. Nat Protoc.

